# Use of GFCF Diets in Children with ASD. An Investigation into Parents’ Beliefs Using the Theory of Planned Behaviour

**DOI:** 10.1007/s10803-019-04035-8

**Published:** 2019-06-05

**Authors:** Rachel E. F. Marsden, John Francis, Iain Garner

**Affiliations:** 1 Department of Psychology, Sheffield Hallam University, Heart of the Campus, Collegiate Crescent, Sheffield, S10 2BP England, UK; 2 Sheffield Hallam University, Unit 2 Science Park, City Campus, Sheffield, S1 1WB England, UK; 3 Sheffield Institute of Education, Sheffield Hallam University, Charles Street Building, Sheffield, England, UK

**Keywords:** GFCF, TPB, ASD, Interventions, Anticipated regret, CAM, Expectancy-value model

## Abstract

Gluten free/Casein free (GFCF) diets are one of the most common types of Complementary and Alternative Medicines (CAM) used in Autism Spectrum Disorders (ASD) despite little evidence to support positive effects. There has been no theory driven literature that has investigated parent’s reasons for their use. The Theory of Planned Behaviour (TPB) was used to examine parent’s intentions to use GFCF diets for their child with an ASD. Treatment and causal beliefs were also examined. Parents (n = 33, children aged 3–17 years) were influenced by anticipated regret, positive outcomes and attitude. Future interventions should provide information to parents and health professionals about the possible causes of ASD and therapy options which are in line with current recommendations.

## Introduction

Higher rates of CAM use are reported in children with ASD compared to typically developed (TD) children (e.g. Christon et al. 2010; Senel 2010; Wong and Smith 2006; Wong 2009), with Gluten Free/Casein Free (GFCF) diets and diet supplements being the most common (e.g. Carter et al. 2011; Christon et al. 2010; Green et al. 2006; Hall and Riccio 2012; Hanson et al. 2007; Pillsbury Hopf et al. 2016). Younger children with ASD and those with more severe symptoms are reported to have higher rates of CAM use (Hall and Riccio 2012; Hanson et al. 2007; Perrin et al. 2012). Parents’ own use of CAM may also be a predictive factor (Hall and Riccio 2012; Wong and Smith 2006; Wong 2009). Parents may be more willing to use CAM when they believe that the CAM treatment has few side effects, high face validity and works on underlying causal mechanisms (Esch and Carr 2004).

CAM use in ASD is used for a variety of reasons, including treating symptoms of autism (35%), concentration/attention (19%), relaxation (23%), GI problems (15%), sleep problems (12%) and communication/speech (12%) (Wong and Smith 2006). Senel (2010) found that parents using special diets for their child rated improvements in behaviour and communication. Within the Chinese population, Wong (2009) reported that 32.5 per cent of parents of children with ASD believed CAM would improve their child’s quality of life. Comparisons between countries can, however, be compromised by cultural background and attitudes (Wong 2009).

When making decisions on whether to use CAM for children with ASD, parents may consider the recommendations from friends, family, parents, medical professionals and the media (Senel 2010; Wong 2009; Wong and Smith 2006). Cornish (2002) noted that parents who were using the GFCF diet for their child had sought information from media sources, support groups and family, more so than from medical professionals. The influence of media sources and parents/family members may be concerning as this information may be biased and incorrect. Using the internet for gaining health information may lead to misdiagnoses or jeopardise the relationship between healthcare practitioners (e.g. Eysenbach and Diepgen 1998; Silberg et al. 1997). Walji et al. (2004) assessed the information presented on CAM related websites and reported that the information presented may cause harm, including avoiding conventional therapy, presenting information on products that may be toxic and almost all websites did not present information on vital warnings, drug interactions or adverse reactions. Walji et al. (2004) however, limited their web search to three herbal therapies and individuals seeking information may use multiple information sources (Walji et al. 2004).

Parent’s causal beliefs about ASD have also been reported to influence decisions about treatment use (Al Anbar et al. 2010). External beliefs about the causes of ASD (e.g. environmental factors; diet, germs) and hereditary attributions were associated with using special diets and vitamin supplements. Dardennes et al. (2011) secondary analysis of the study reported that parents had stronger causal beliefs about ASD that were attributed to brain abnormalities followed by genetic factors. Furthermore, stronger beliefs in the etiological role of food allergy and a chemical imbalance was associated with higher use of detoxification treatments, diets, vitamins and reduced drug use. Children’s and parents age was not related to any CAM treatment, suggesting that causal beliefs are stable over time (Dardennes et al. 2011).

Parents who do not use GFCF diets or discontinue use may believe they have greater control over their child’s symptoms and behaviour; increased personal control has been associated with reduced use of diets and vitamin supplements (Kuppin and Carpiano 2006). Those who believe that they would not have social and/or medical practitioner support, and value their approval for the use of CAM, may also be less likely to use CAM (Conner and White 2009). Wong and Smith (2006) reported that many parents of children with ASD and those with TD children did not inform their physician or paediatrician about their CAM use. Increased cost and time may also predict non-use. Christon et al. (2010) reported that 44.7 per cent of parents found the cost of CAM treatment difficult to meet, leading to discontinued use in 17.8 per cent of cases. Most parents reported that the time expenditure necessary for CAM treatment was easy, however, 20 per cent found that the time expenditure was difficult to meet. As results were across all types of CAM, Christon et al. (2010) stated that different treatments yield different results.

The decision to use CAM may be influenced by the value placed on the treatment (Christon et al. 2010). Parents who invest more time and money in treatments may be more likely to continue with the treatment (Mudford et al. 2000) and may perceive greater benefits for their child. This, in turn, may magnify placebo effects on the efficacy of the treatment. Cornish (2002) reported that a small minority of those who discontinued GFCF diets did so due to perceiving no noticeable changes in behaviour, cost implications, social reasons and palatability and restricted choice of substitute foods. Parents who remain on GFCF diets may also have children who accept a wider variety of foods (Cornish 2002).

Although previous studies may involve selection biasing (e.g. Wong and Smith 2006), over-representing of children with chronic disease (Davies and Darden 2003), recruitment of predominantly white middle- to upper-class respondents which may account for high prevalence rates, particularly as increased CAM use is related to higher education and income (Hall and Riccio 2012; Hanson et al. 2007), a main limitation is the lack of theory-driven literature on children’s CAM use (Robinson et al. 2009). There is currently no theory-driven literature investigating reasons for GFCF diet use. Failing to utilise a theoretical framework can jeopardise the reliability and validity, along with the generalisability of the explanations for decision-making (Lorenc et al. 2009). Using an existing model allows methodological consistency (Robinson et al. 2009).

**Fig. 1:**
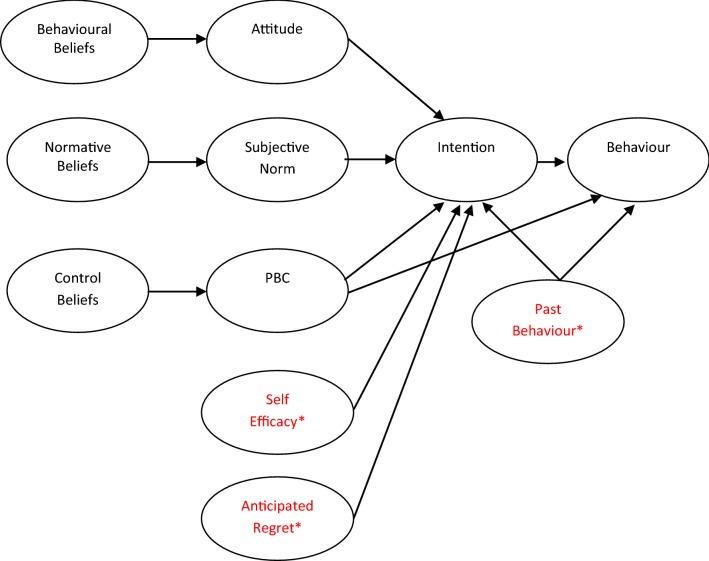
Theory of Planned Behaviour: predictors of intentions and behaviour. *Variables in red depict extensions

The Theory of Planned Behaviour (TPB) (Ajzen 1988, 1991), a widely accepted theory within Health Psychology, aims to account for psychological influences and explain individual differences in behaviour (Lorenc et al. 2009). The TPB states that when an individual has stronger Intentions to engage in a particular behaviour (e.g. exercise regularly) they are more likely to actually engage in the behaviour (Ajzen 1991). The TPB suggests that Intentions are influenced by the underlying attitudes, subjective norms and perceived behavioural control (PBC) and all can be measured directly and quantitatively (Fig. 1). Where attitudes are the person’s evaluation of possible outcomes of the intended behaviour, subjective norms are the persons perceived social pressure from others to engage with the behaviour. PBC is defined as the person’s perception of control over the intended behaviour (Ajzen 2002a). The major constructs of attitude, subjective norms and PBC can also be measured indirectly from a set of corresponding beliefs (Ajzen 2002a); behavioural, normative and control beliefs. Behavioural beliefs are the person’s beliefs about the possible outcome combined with the evaluation of the outcome; these in turn formulate attitudes. Normative beliefs are the person’s perceived social pressure to engage with the behaviour combined with their motivation to comply with significant others; these formulate subjective norms. Finally, control beliefs are the person’s perceptions about control over barriers or facilitators of the intended behaviour; these formulate PBC (Armitage and Conner 1999b). The TPB suggests that the more favourable the attitude, subjective norm and greater the PBC, the stronger an individual’s intention will be (Ajzen 2002a). Past behaviour, anticipated regret of either performing the behaviour or not, and self-efficacy (SE; the belief in one’s own ability) commonly extend the model. Although there has been debate into the independent predictive power of SE from PBC, some argue for their distinction (Armitage and Conner 1999a; Furnham and Lovett 2001).

As the TPB focuses on specific cognitions which affect responses, it is particularly suitable for CAM research as CAM use is often based upon individual, specific choices (Lorenc et al. 2009). Using the TPB, Hirai et al. (2008) reported that patients decisions on whether to use CAM was influenced by family pressure. Furnham and Lovett (2001) reported that the TPB was a successful application to the prediction of intentions to use homeopathy. Stronger intentions to use homeopathy were associated with more positive attitudes towards homeopathy and higher levels of PBC and ability. Furnham and Lovett (2001) suggest that the belief that homeopathy reduces symptoms was surprising due to the lack of empirical evidence for its effectiveness. Conner and White (2009) also demonstrated the utility of the TPB whereby intenders to use CAM were more likely to believe in positive consequences of CAM.

The TPB has successfully discriminated between users and non-users of dietary supplements (Conner et al. 2001). Users displayed stronger intentions, positive attitudes, perceived more normative pressure (social pressure from significant others) and greater PBC towards supplements. Attitude was the strongest predictor whereby users of supplements believed that they would ‘stop them getting ill’ and ‘help them be healthy’ (Conner et al. 2001). Conner et al. (2001) study also revealed the powerful nature of media on decision-making. Similarly, Furnham and Lovett (2001) found that family and friends were important factors whereby those with stronger intentions to use CAM had stronger motivations to comply with family and homeopathic practitioners and a weaker motivation to comply with GPs. Within Conner and White (2009) investigation, family members were the most dominant predictor of subjective norms and intenders were more likely to believe that other family members, peers and medical professionals would approve of their use of CAM. The TPB has provided further information on various control factors which may influence CAM use. Furnham and Lovett (2001) reported that those with stronger intentions to use CAM were less likely to believe that homeopathy ‘is expensive’, ‘takes too much time’ and ‘requires confidence’. Subjective norms however were a stronger predictor of intention compared to PBC, suggesting the importance of social approval (Furnham and Lovett 2001). Conner and White (2009) concluded that CAM use is more likely when people believe there to be health benefits, perceive the support of significant others and are not dissuaded by potential barriers.

Ajzen (2002b) proposed that people often have intentions to perform behaviour but this does not always predict actual behaviour that is followed through. Experience and the frequency of past behaviour can often predict future behaviour more reliably when compared to the other variables of the TPB (e.g. intentions) (Conner and Sparks 2005). Ajzen (2002b) suggests that if an individual has unrealistic beliefs towards performing a behaviour and intentions are unstable, their beliefs may not clearly guide behaviour. Past behaviour may therefore act as better predictor for future behaviour.

When parents are making decisions on whether to use GFCF diets for their child with ASD, they may also consider factors relating to their own ability to administer the diet. SE’s ability to predict people’s intentions has shown mixed evidence when applied to CAM use, but does emerge as a distinct variable to PBC (Furnham and Lovett 2001). Implementing GFCF diets for children with ASD may be perceived as onerous and time-consuming therefore parents own SE may influence the implementation of CAM.

Other factors such as internal and external Locus of Control (LoC) can influence our behaviour and may relate to CAM use (e.g. Furnham and Kirkcaldy 1996; Sasagawa et al. 2008). Locus of Control refers to one’s belief that certain outcomes are a result of our own behaviour or traits (internal) or as a result of other forces or due to chance (external) (West et al. 2018). Findings on LoC are however mixed (e.g. Furnham and Forey 1994; Sirois and Gick 2002) and may be due to a wide range of health-related problems between participants, including CAM users, who had more medical and chronic health problems than conventional medicine users. Both chronic pain and an increased number of health problems are associated with less self-control (Crisson and Keefe 1988). Furnham and Beard (1995) suggest that internal and external beliefs are not always mutually exclusive, and an individual can represent both beliefs within the same context. The literature on LoC is mixed with variants in differences between measures and within item and subscale analysis. In addition, the literature varies between the types of CAM used and the medical problems that patients are seeking CAM for. Surette et al. (2011) note that variances in CAM definitions may result in different classifications of CAM thus leading to different beliefs. Finally, anticipated regret, may also improve the predictions of intentions (Newton et al. 2013). Anticipated regret has, to date, not been examined within the area of CAM use and GFCF diet use in ASD.

### Current study

Many previous studies have not used structured questioning to determine what types of CAM are being used for children with ASD and the underlying reasons for this, including causal beliefs about ASD (Surette et al. 2011; Wong 2009). CAM users are not a homogenous group and research should identify determinants of particular types of CAM (Hendrickson et al. 2006). It remains unclear as to why parents of children with ASD use GFCF diets when there is little evidence to support their positive effects and may carry associated risks (Arnold et al. 2003; Hediger et al. 2007). The TPB is a widely used model for understanding various health behaviours and it’s suitability for understanding behaviour related to CAM use has been demonstrated (e.g. Conner and White 2009; Furnham and Lovett 2001). In addition many of the factors within the TPB may be particularly important for understanding parents use of CAM for their child with ASD (e.g. Christon et al. 2010; Senel 2010). This present study utilises an online TPB questionnaire to investigate parents’ beliefs and factors that may predict intentions to use GFCF diets for their child with ASD. A range of hypotheses were predicted including: users of GFCF diets will indicate higher scores on the TPB variables compared to non-users (e.g. increased positive *attitudes* towards the perceived benefits of the diet, increased *PBC*, increased perception of social pressure to use the diet (*subjective norms*) and increased *intentions* to use the diet); causal and treatment beliefs about ASD will differ between users and non-users, with users indicating stronger beliefs towards dietary treatment; *attitude*, *PBC*, *SE*, *subjective norms* and *anticipated regret* will be positively correlated with *intentions.* Further exploratory analyses were also conducted.

## Method

### Participants

Seven organisations took part and advertised the online study to prospective parents with children with ASD. Thirty-three parents who had children with ASD (parental report) participated in the study examining parents’ beliefs about GFCF diets for the treatment and management of their child’s symptoms of autism and causal and treatment beliefs. Demographics for children are presented in Table 1. Twenty-one parents indicated that their child had other diagnosed conditions (ADHD = 7, epilepsy = 3, Tourette syndrome = 2, dyspraxia = 4, sensory processing disorder = 5). Parents gave details of their own use of CAM and previous use of GFCF diets for their child (Table 2).

**Table 1:** Demographics of children

	Children
N	33
Age	
Mean	10 yrs
SD	3.34
Range	3–17
Gender	23 males, 10 females
Diagnosis (N)	
Autism	15
Asperger	6
ASD	11
PDD-NOS	1
Age of diagnosis	
Mean	5 yrs
SD	2.57
Range	2–11

**Table 2:** Parent Use of CAM and use of GFCF Diets for their Child

	CAM	GFCF diets
Never used	10	25
Currently use	7	8 (current and past use)
Past use	6	
Considering using	3	9
Planning to use	–	3

### Elicitation Study

In accordance with the recommendations put forth by Ajzen and Fishbein (1980) and Ajzen (2002a) an elicitation study was conducted to elicit parents’ modal behavioural, normative and control beliefs related to using GFCF diets for the treatment and management of their child’s symptoms of ASD. Questions from the Multidimensional Health Locus of Control scale (MHLoC; Wallston, Kaplan, and Maides, 1976) were added to the questionnaire to elicit further beliefs about the possible outcomes of the GFCF diet. Using an online questionnaire, twenty-six parents (recruited independently from the main study) with children with ASD (5–15yrs, M = 8.6, SD = 2.7) were asked to list the advantages and disadvantages of using a GFCF diet (*behavioural beliefs*), identify which groups of people would approve and disapprove of their use of GFCF diets (*normative beliefs*) and which factors and conditions would make it easy or difficult to implement a GFCF diet (*control beliefs*). Responses were content analysed and beliefs with similar semantic outcomes were grouped as one salient belief (category). Inter-rater agreement was achieved on both the categories and the frequencies of responses within categories.

The most frequently rated *behavioural beliefs*, *control beliefs* and salient referents (*normative beliefs*) elicited formulated the modal set of *behavioural*, *normative* and *control* beliefs in the TPB questionnaire. Categories that were not frequently mentioned, yet were common findings in the literature (e.g. cost of the GFCF diet) were also included. Fourteen types of salient positive and negative *behavioural beliefs* were elicited and grouping resulted in nine belief outcome categories (diet variety was categorised as worsened and improved) (Table 3). Twelve salient *control beliefs* were elicited resulting in 10 salient control belief categories and five individual salient referents were elicited and formulated the *normative beliefs* (Table 4).

**Table 3:**
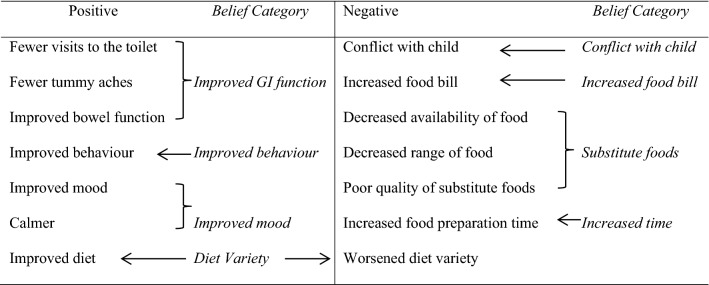
Elicited salient positive and negative outcome beliefs (Behavioural Beliefs)

**Table 4:** Elicited salient Control Beliefs and Normative Referents

Control beliefs	Salient referents
Availability of substitute foods	School/teachers
Increased cost of substitute foods	Professionals (educational, medical, other)
Taste of substitute foods	Family
Increased cost of food bill	Other parents
Availability of substitute foods when eating out	Friends of the parent
Child’s difficulty in adapting to changes in routine/structure/importance of routine/structure	
Sensory problems of the child	
Environmental factors (sensory overload)	
Mood of the child	
Amount of sleep of the child	

### Main TPB Questionnaire

The TPB questionnaire was created following standard procedures (Ajzen 1991, 2002a; Ajzen and Fishbein 1980; Conner and Sparks 2005). Questions were modified for the appropriateness of this study in accordance with previous literature (Conner and Sparks 2005; Conner and White 2009; Furnham and Lovett 2001). Predictor variables (*attitude, subjective norm, PBC, anticipated regret, SE)* for *intentions* were assessed directly using standard questioning (Conner and Sparks 2005; Conner and White 2009; Furnham and Lovett 2001). *Attitude, subjective norm* and *PBC* were assessed indirectly through questions based upon beliefs from the elicitation study.

### Measures

#### Intention

Parents’/caregivers’ intention to use GFCF diets in the next 12 months was assessed using four questions each on a seven-point Likert scale ranging from extremely unlikely to extremely likely; ‘I intend to use GFCF diets for the treatment and management of my child’s symptoms of ASD in the next 12 months,’ ‘I want to use GFCF diets for the treatment and management of my child’s symptoms of ASD in the next 12 months,’ ‘I plan to use GFCF diets for the treatment and management of my child’s symptoms of ASD in the next 12 months,’ and ‘I will use GFCF diets for the treatment and management of my child’s symptoms of ASD in the next 12 months’. Conner and Sparks (2005) recommend the use of multiple items for *intentions* to increase the measure’s reliability. Each item was scored on a bipolar scale (−3 to +3) and intention to use GFCF diets was assessed as the mean of the four items (composite score), with higher scores representing stronger *intentions* to use GFCF diets in accordance with Conner and Sparks (2005); Cronbach’s alpha was 0.992.

#### Attitude

In accordance with Furnham and Lovett (2001) and recommended by Ajzen (2002a) and Conner and Sparks (2005), both direct and indirect measures of *attitude* were taken. The direct measure of *attitude* was established using a semantic differential scale. Parents were provided with the sentence ‘my using GFCF diets for the treatment and management of my child’s symptoms of ASD in the future would be…’ and given seven pairs of adjectives to be rated on a seven-point Likert scale: good/bad, harmful/beneficial, pleasant/unpleasant, foolish/wise, enjoyable/unenjoyable, unnecessary/necessary and worthless/valuable. Both instrumental and experiential (affective) aspects are recommend to be used, as *intentions* may be related to affective aspects rather than cognitive measures (Ajzen 2002a; Conner and Sparks 2005). The positive and negative end points were counterbalanced to counteract possible response sets (recommend by Ajzen 2002a). Each of the seven scales were scored on a bipolar scale (−3 to +3) (reversing items reflecting lower scores for a positive attitude towards GFCF diets) and the mean of the seven items were taken as the composite score. Cronbach’s alpha of the direct measure was 0.96.

The indirect measure of *attitude* (belief-based measure) was taken from *behavioural beliefs* derived from the elicitation study. Parents rated both the likelihood (extremely unlikely/extremely likely) and evaluated the outcome (extremely bad/extremely good) on a seven-point Likert scale and scored using a bipolar scale (−3 to +3) to give a belief-based measure of attitude as recommend by Ajzen and Fishbein (1980), Conner and Sparks (2005) and Ajzen (2002a). Each *behavioural belief* was multiplied by the corresponding outcome evaluation and these products were summed to give an indirect measure of attitude. Ajzen and Fishbein (1980) notes that *attitudes* are based on both positive and negative consequences of the behaviour in question. *Attitudes* correspond to the favourability or unfavourability of the total set of these outcomes, with each one weighted by the measure of strength that the person’s believes that performing the behaviour will lead to the outcome. This is known as the expectancy to value model of *attitude*. Ajzen and Fishbein (1980) highlights that by measuring the likelihood and value of the outcome takes into consideration the importance of the outcome, which can be evaluated positively or negatively. Ajzen (2002a) also states that a bipolar scale is essential to assess the strength of beliefs. When an individual disagrees that behaviour will lead to a negative outcome this contributes positively to the *attitude*. Therefore, unlike a unipolar scale, a bipolar scale treats a disbelief in a negative outcome as ‘not believing’ the behaviour will lead to that outcome and assumes the person would therefore have a positive evaluation of the behaviour *not* having the negative outcome (Ajzen and Fishbein 1980). Cronbach’s alpha of the summed score was 0.72.

#### Subjective Norm

The direct measure of *subjective norm* was derived from the composite score of three items. Measures of *subjective norm* should include injunctive normative influences (what significant others think the person should do) and descriptive normative influences (what significant others are perceived to do) (Conner and Sparks 2005). Conner and Sparks (2005) also noted that, although *subjective norms* are typically weaker at predicting *intentions* compared to other TPB variables, those studies that had included multi-item measures of *subjective norms* were stronger predictors of *intentions* (although still weaker than *PBC* and *attitudes*). Injunctive norms included ‘the people in my life whose opinion I value think I should implement a GFCF diet for the treatment/management of my child’s symptoms of autism’ (rated from strongly disagree to strongly agree), ‘the people in my life who’s opinion I value would…(approve/disapprove) of my using GFCF diets for the treatment/management of my child’s symptoms of autism’ (reversed scored) and the one descriptive norm was ‘most of my friends with children with an ASD implement the GFCF diet for their child for the treatment and management of ASD symptoms’ (rated from strongly disagree to strongly agree). Each item was rated on a seven-point Likert scale and scored on a seven-point unipolar scale from +1 to + 7. The mean score of the direct measure of *subjective norm* was used with higher scores indicating more perceived pressure to use GFCF diets. Cronbach’s alpha coefficient for the three items was 0.72.

The indirect measure of *subjective norm* was derived from *normative beliefs* and the motivation to comply with the referent. Parents were asked to indicate on a seven-point scale ranging from extremely unlikely to extremely likely the likelihood that salient others think they should use a GFCF diet (how likely is it that (salient referent) thinks that you should use GFCF diets for the treatment/management of your child’s symptoms of autism?). Each *normative belief* was weighted against parents’ motivation to comply with the referent (With regard to my use of GFCF diets for the treatment/management of my child’s symptoms of ASD, I want to do what (salient referent) thinks I should do). *Subjective norm* can then be calculated using the expectancy-value formula (Ajzen 2002a), by multiplying normative beliefs with motivation to comply and summing the products (Ajzen and Fishbein 1980). By accounting for motivation to comply the important referents are given more weight in the prediction of *subjective norm* (Ajzen and Fishbein 1980). In accordance with Ajzen and Fishbein (1980) and Conner and Sparks (2005) normative beliefs were scored on a bipolar scale (strong negative pressure to perform: −3, to a strong positive pressure to perform: +3) and motivations to comply were scored on a unipolar scale (1 to 7) as people are unlikely to be motivated to do the opposite of what their salient referents think they should do (Ajzen and Fishbein 1980; Conner and Sparks 2005). This scoring method allows a negative belief (e.g. −2) that a salient referent thinks that they should engage with the behaviour in question when multiplied by a strong motivation to comply (e.g. +7) gives an overall score of −14, which represents the negative *subjective norm* (unfavourable social pressure). Cronbach’s alpha for the summed scale was 0.65.

#### PBC and Self Efficacy

The direct measure of *PBC* was assessed using four items, which parents rated on a seven-point scale. These were, ‘Whether or not I use GFCF diets for the treatment and management of my child’s symptoms of ASD is entirely up to me (strongly disagree/strongly agree)’, ‘How much personal control do you feel you have over using GFCF diets in the next 12 months (very little control/complete control)’, ‘I believe I have the resources to implement a GFCF diet for the treatment/management of my child’s ASD symptoms (true/false: item reversed)’, and ‘For me to implement a GFCF diet on my child in the future would be…(impossible/possible)’. Ajzen (2002c) argued that *PBC* can be considered as a second order construct and consists of two components: *SE* and perceived controllability. *SE* involves the ease or difficulty of performing a behaviour, including confidence (Ajzen 2002a). Ajzen (2002c) suggests that *SE* can be elicited using difficulty and perceived confidence. The *PBC* component involves individuals’ beliefs of control over the behaviour and that the behaviour is up to them. Conner and Sparks (2005) highlight that there is no agreement of whether *PBC* component of the TPB should be a unidimensional measure, combining both perceived controllability and *SE* or whether it would be better to examine the predictive power of these two components separately. However, several authors noted a distinction between *SE* and perceived control (e.g. Armitage and Conner 1999a, b; Furnham and Lovett 2001; Norman and Hoyle 2004), thus within this study the bidimensional nature of the *PBC* will consider these two components separately in their predictions for *intentions*. It is also argued that *PBC* can predict behaviour directly, whereas *SE* can only predict behaviour via *intentions* (Norman and Hoyle 2004). Questions relating to *SE* were constructed in accordance with Furnham and Lovett (2001) and included ‘I am confident that I can implement a GFCF diet for the treatment/management of my child’s ASD symptoms (true/false: item reversed)’, ‘I believe I have the ability to use GFCF diets for the treatment/management of my child’s symptoms of ASD (definitely do not/definitely do)’, and ‘To what extent do you feel yourself capable of using GFCF diets (not at all capable/extremely capable)’. Both *PBC* and *SE* were scored on a seven-point unipolar scale. The mean of the items measuring *PBC* and *SE* provided a composite score for each, with higher scores represented a higher perceived control and ability to use GFCF diets. Cronbach’s alpha were 0.59 for PBC and 0.87 for SE.

The indirect measure of *PBC* was derived from measures of *control beliefs* and the power of these factors to facilitate the use of GFCF diets. *Control beliefs* assess the presence or absence of factors that may facilitate or inhibit the behaviour and are commonly scored from never to frequently, false to true, unlikely to likely (Conner and Sparks 2005). The 10 *control belief* categories were inputted into the questionnaire, e.g. ‘there is a lack of availability of substitute foods for a GFCF diet (never/frequently)’. An example of a corresponding power item is ‘a lack of availability of substitute foods makes my use of the GFCF diet…(less likely/more likely)’. These were rated on seven-point Likert scales.

Conner and Sparks (2005) note that when using response formats of never to frequently a unipolar is more appropriate. Ajzen (1991) notes that the scoring of the power items is unclear, thus wording of response formats should guide the use of bipolar or unipolar (Conner and Sparks 2005). *Control beliefs* (frequency of occurrence) were scored on a unipolar scale (1 to 7) and the corresponding power items were scored on a bipolar scale (−3 to +3). The scoring procedure was in accordance with the recommendations from Conner and Norman (2005) and Ajzen (1991). These were then multiplied by the perceived power (expectancy-value model) of the factors to facilitate or inhibit the use of GFCF diets (rated from less likely to more likely) and summed to provide a composite score for an indirect measure of *PBC*. This scoring technique of a unipolar and bipolar scale allows for composite scores to reflect both positively and negatively towards control factors, whereby a negative control score will be unfavourable to the use of a GFCF diet and positive scores will represent a favourable control belief in the use of GFCF diets. Cronbach’s alpha was 0.87.

#### Past Behaviour

*Past behaviour* considered both parents’ current/past use of CAM for the treatment/management of conditions they may have, and stated what they had used, as well as whether they were considering the use of CAM and their past/current use of the GFCF diet for their child. Parents indicated on a yes/no response format on whether they were thinking about and planning on using a GFCF diet in the near future. In addition, parents stated whether other family and friends had used CAM and GFCF diets.

#### Anticipated Regret

Questions relating to *anticipated regret* were adapted from Newton et al. (2013) and Abraham and Sheeren (2004). *Anticipated regret* was measured using three items: ‘If I did not implement a GFCF diet as a treatment/management of symptoms of ASD for my child I would regret it’, ‘If I did not implement a GFCF diet as a treatment/management of symptoms of ASD for my child it would bother me’ and ‘I would be disappointed in myself if I did not implement a GFCF diet as a treatment/management of symptoms of ASD for my child’ (rated on a seven-point unipolar scale from strongly disagree to strongly agree). Items were scored on a unipolar scale and the mean of the three items provided the composite score; Cronbach’s alpha was 0.89. Higher scores represented a favourable response to the GFCF diets and increased regret in not using a GFCF diet.

#### Lay Beliefs about Autism Questionnaire (LBA-Q, Furnham and Buck 2003)

The LBA-Q explores beliefs of the causal factors and treatment of autism. The questionnaire contains 24 statements related to the aetiology and treatment of autism scored on a seven-point Likert scale (very inaccurate/very accurate). Five factors have been identified which account for 54 per cent of the variance (Dardennes et al. 2011). The factors are, *psychogenic and external* (internal reliability, 0.82), which relate to upbringing, luck and God, *pregnancy and environmental* (internal reliability, 0.61), which includes problems during pregnancy and the helpful role of others, *genes and drugs* (internal reliability, 0.67), *diet* (internal reliability, 0.74) and *brain abnormalities* (internal reliability, 0.59). The mean of the total scores within each factor provided a score, with higher scores indicting positive beliefs.

### Analysis

A range of non-parametric analyses were performed to examine hypotheses. *Intentions* to use a GFCF diet in the next 12 months was the criterion variable for the regression analyses. A logistic regression was the most suitable method for analyses. *Intentions* were coded as 0 (low intention = <0) and 1 (high intentions =>0).

## Results

Participants were characterised according to *users* and *non*-*users* of GFCF diets. *Users* included all participants who were currently implementing a GFCF diet and those who had used GFCF diets in the past. *Intentions* to use a GFCF diet in the next 12 months was the criterion variable for regression analyses.

### Differences Between Users and Non-Users on the TPB Variables

Differences between *users* of GFCF diets for their child and *non*-*users* on each of the components of the TPB were examined (Table 5). *Users* indicated increased positive *attitudes*, *PBC* and *SE* over using GFCF diets. *Users* also showed positive outcome beliefs (*behavioural beliefs*) about using GFCF diets as indicated by the positive scores above zero. In contrast, *non*-*users* indicated negative outcome beliefs about using the GFCF diet (scores below zero). *Non*-*users* also had significantly stronger beliefs about factors that may prevent them using GFCF diets *(control beliefs)*. Both *users* and *non*-*users* had scores below zero for *normative beliefs*, indicating lower perceived pressure to use GFCF diets from significant others.

**Table 5:** Comparison of Users and Non-Users on TPB Variables

Variable	Non-users (n = 25) users (n = 8)		U	Z	P (1-tailed)
	Median				
Intentions	−3	3	47.0	−2.35	p = 0.03
Attitude	−2.9	3	34.5	−2.76	p = 0.01*
Subjective norm	−2.6	5	52.5	−2.0	p = 0.04
PBC	4.7	6.4	42.5	−2.43	p = 0.01*
SE	4.33	6.7	26.0	−3.14	p = 0.001*
Anticipated	2	6	47.0	−2.27	p = 0.02
Regret					
BB	−31	12.5	28.0	−3.03	p = 0.004*
NB	−14	-0.5	39.5	−2.55	p = 0.01*
CB	−82	0	21.0	−3.32	p = <0.01*

Independent Mann–Whitney U Tests were performed

*BB* Behavioural beliefs, *NB* Normative beliefs, *CB* Control beliefs

*Due to multiple testing, a more stringent probability value of 0.01 was used

### Differences the LBA-Q Variables

The mean scores for the whole sample of the five factors of the LBA-Q are presented in Table 6. A repeated measures ANOVA revealed a significant difference between mean scores (F(3, 98) = 25.80, p = <0.001) (Greenhouse–Geisser correction applied). An overall effect size of 0.446 suggests that 45% of the variation in scores can be accounted for by differing means between the five factors. Overall parents’ beliefs in the treatment and causes of ASD fall within the factors *genes and drugs, diet* and *brain abnormalities*. Considering the scale of measurement for factors was between one and seven, mean scores do not reflect high ends of the scale, suggesting uncertainty in beliefs.

**Table 6:** Mean Scores and Standard Deviations From the Five Factors of the LBA-Q

Factor	Mean	SD
Psychogenic and external	1.55	0.67
Pregnancy and environmental	2.65	1.21
Genes and drugs	3.38	1.08
Diet	3.50	1.54
Brain abnormalities	3.9	1.55

### Regression Analysis on the Causal Beliefs about ASD

Further exploratory analysis examined the relationship between casual beliefs about ASD and *intentions* to use a GFCF diet. A logistic regression with *intentions* as the criterion variable and factors of the LBA-Q as predictor variables (overall model significant, p = 0.006, with 75% of cases identified correctly) indicated that *diet* was the only factor associated with higher *intentions*. A higher belief in *diet* causes of autism was significantly associated with higher *intentions* to use GFCF diets (Chi square (5) = 16.37, p = 0.01). An odds ratio of 4.52 (CI (95%), 1.41, 14.48) suggests that for every one unit increase in a belief in diet causes, the odds of higher *intentions* to use GFCF diets was 4.52 times more likely. The R-squared value of 0.56 indicated that 56 per cent of the variance could be accounted for by the predictors.

Furthermore, a logistic regression, with *users* as the criterion variable and factors of the LBA-Q as predictor variables (model significant p = 0.004; 76 per cent of cases identified correctly) indicated that an increase in beliefs about *diet* and *pregnancy and environmental* was associated with *users* of GFCF diets (*diet*, Chi square(5) = 17.41, p = 0.02, and *pregnancy and environmental*, Chi square(5) = 17.41, p = 0.02). An odds ratio of 4.49(CI (95%), 1.33, 15.11) for *diet* suggests that for every one unit increase in a belief in *diet* the odds of using GFCF diets was 4.49 times more likely. An odds ratio of 7.56 (CI (95%), 1.46, 39.24) for *pregnancy and environmental* suggests that for every one unit increase in a belief in *pregnancy and environmental* the odds of using a GFCF diet is 7.56 times more likely. Sixty-seven per cent of the variance could be explained by the predictor variables.

### Correlational Analysis Between Variables of the TPB

Relationships between the TPB variables were examined, specifically whether *attitudes*, *PBC*, *SE*, *subjective norm* and *anticipated regret* were positively correlated with *intentions,* and whether indirect measures (*behavioural*, *normative* and *control beliefs*) were positively correlated with the respected *attitude*, *subjective norm* and *PBC*. As data was non-parametric, Spearman’s r is reported (Table 7). Due to multiple correlations and the risk of type 1 errors, only correlations significant at the 0.01 level are considered. The only indirect measure to correlate with its direct predictor was *behavioural beliefs* with *attitude*. *Attitude* (both direct and indirect measure), *subjective norm* (direct and indirect measure) and *anticipated regret* were highly correlated with *intentions* to use GFCF diets in the next 12 months. This indicates that higher *intentions* to use the diet were related to increased positive *attitudes* about the diet, an increased perceived pressure to use GFCF diets from significant others (*subjective norm*) and increased feelings of *anticipated regret* if they were not to use GFCF diets.

**Table 7:** Correlations between Measured Variables of the TPB

	Intention	Attitude	Subjective norm	PBC	SE	Anticipated regret	Behavioural beliefs (BBxOE)	Normative beliefs (NBxMC)	Control beliefs (CBxP)
Intention		0.701*	0.660*	0.011	0.076	0.738*	0.465*	0.521*	−0.111
Attitude			0.610*	−0.346	−0.60	0.578*	0.731*	0.422	0.035
Subjective norm				−0.130	−0.086	0.674*	0.582*	0.417	0.005
PBC					0.845*	−0.111	−0.320	−0.072	0.079
SE						−0.057	−0.091	−0.05	0.354
Anticipated regret							0.442*	0.430	0.383
Behavioural beliefs (BB × OE)								0.430	0.383
Normative beliefs (NB × MC)									0.035

Spearmans’ r. correlations

*BBxOE* behavioural beliefs multiplied by outcome evaluation; *NBxMC* normative beliefs multiplied by motivation to comply; *CBxP* control beliefs multiplied by power

*Correlations are significant at the 0.01 level (1-tailed)

*PBC* was not correlated with *intentions* but was highly correlated with *SE,* indicating that higher perceived control was related to higher perceived ability to use the diets. A*nticipated regret* was positively correlated with *behavioural beliefs,* suggesting that those who display feelings of regret of not using GFCF diets have more positive outcome beliefs. Although higher *normative beliefs* were positively correlated with *intentions* to use GFCF diets, *users* and *non*-*users* had inhibiting beliefs about using GFCF diets.

### Regression Analysis between Intentions and TPB Variables

A series of logistic regressions were performed. At the first step, direct measures of *intentions* were entered (*attitudes*, *subjective norm*, *PBC*, *SE* and *anticipated regret)* to analyse the hypothesis that they will be associated with higher *intentions.* At the second step the indirect measures of *attitude*, *subjective* norm and *PBC* were entered to examine whether more positive beliefs, and stronger perceived pressure and control are associated with higher *intentions*. An increase in *anticipated regret* was significantly associated with those who had higher *intentions* to use a GFCF diet within the next 12 months (Chi square (5) = 27.48, p = 0.05). An odds ratio of 4.14 (CI (95%), 1.00, 17.41) suggests that for every one unit increase in *anticipated regret* the odds of having higher *intentions* was 4.14 times more likely. An R-square of 0.80 suggests that 80 per cent of the variance can be explained by the predictors. Collinearity was not detected. In step two of the regression analysis *anticipated regret* was no longer significant, indicating that the indirect measures of *attitude*, *subjective norm* and *PBC* may mediate the role of *anticipated regret*.

### Regression Analysis between Users and Non-Users and TPB Variables

A logistic regression was performed using the method by Conner et al. (2001). At the first step *intentions* and *PBC* were entered, at the second step *attitude*, *subjective norm* and *anticipated regret* were entered and at the third step, age and indirect measures of *attitude*, *subjective norm* and *PBC* were entered. Conner et al. (2001) suggest that this allows the model to test whether the behavioural effects of *attitude*, *subjective norm* and belief components are mediated by *intention* and *PBC* as the TPB predicts. At the first step, *intentions* and *PBC* were a significant predictor of *users* of GFCF diets with 84 per cent of participants classified correctly (*intentions*, Chi square (2) = 6.811, p = 0.02, and *PBC*, Chi square (2) = 16.811, p = 0.05). For *intentions* an odds ratio of 2.10 (CI (95%), 1.11, 3.97) suggests that for every one unit increase in *intentions* the odds of using GFCF diets was 2.1 times more likely. For PBC an odds ratio of 6.42 (CI (95%), 1.04, 39.63) suggests that for every one unit increase in PBC the odds of using GFCF is 6.42 times more likely. At the second and third step no variable was significant, suggesting that the effects of *attitude*, *subjective norm* and *anticipated regret* are mediated by *intentions* and PBC.

### Correlational Analysis between Individual Beliefs and their Direct Measure and Intentions

Further analysis examined Spearman correlations between individual *behavioural*, *normative* and *control beliefs* with *intentions*, *attitude, subjective norm and PBC*. Only one arm of the expectancy-value model was used, using the individual belief-based items *behavioural beliefs*, *normative beliefs* and *control beliefs*. This avoids problems with scaling from multiplying two variables not measured on a ratio scale (Gagne and Godin 2000). It is also commonly demonstrated that using the belief based items only yields higher or similar coefficient correlations to their direct measures (Ajzen 1991; Gagne and Godin 2000; Rhodes et al. 2009). Only those correlation coefficients that were significant at the 0.01 level were considered due to multiple correlations. Table 8 shows that a positive *behavioural belief* related specifically to improved GI symptoms and improved mood was related to increased *intentions* to use the diet. A worsened diet variety and introducing conflict with the child was negatively related, suggesting decreased *intentions* to use the diet. Perceived pressure to use GFCF diets from family, friends and professionals were positively correlated with *intentions*.

**Table 8:** Correlation coefficients of individual beliefs with corresponding direct measures and intentions

	Intentions	Attitude	PBC	Subjective norm
Behavioural beliefs				
Improved GI symptoms	0.595*	0.673*		
Improved mood	0.806*	0.843*		
Worse diet variety	−443*	−0.591*		
Improved diet variety	0.376	0.493*		
Conflict with child	−0.402*	−0.476*		
Increase food bill	−0.21	−0.254		
Increased time	−0.359	−0.391*		
Substitute foods	−0.172	−0.268		
Normative beliefs				
Family	0.667*			0.543*
School/teachers	0.302			0.411*
Other parents	0.112			0.412*
Friends	0.448*			0.544*
Professionals	0.521*			0.441*
Control beliefs				
Lack of availability	−0.070		0.246	
Taste	−0.164		0.372	
Expensive	0.038		0.133	
Increased food bill	0.024		0.239	
Eating out	−0.200		0.104	
Adapting to change	−0.328		0.047	
Sensory problems	0.118		0.037	
Sensitive environment	0.024		-0.071	
Mood changes	0.119		0.009	
Sleep changes	0.283		0.210	

Spearman’s’ r. correlations

*Correlations are significant at the 0.01 level (1-tailed)

Most *behavioural beliefs* about outcome correlated with their direct measure *attitude* (excluding increases in the food bill and issues with substitute foods). Higher positive outcome beliefs in improved GI symptoms, mood and improved diet variety may be related to increases in positive *attitude*. In contrast, increased negative outcome beliefs in worsening diet variety, conflict with child and increased time was related to a decrease in positive *attitudes*. Items of the belief-based measure of *PBC* (*control beliefs*) did not correlate with PBC. All *normative belief* referents were related to increased *subjective norm*, suggesting that increased pressure to use GFCF from significant others increases perceived pressure to use the GFCF diet.

## Discussion

The findings of this study partially supported the hypothesis that users of GFCF diets would have increased positive attitudes and outcome beliefs about using the GFCF diet for their child with ASD. It was also demonstrated that users of the diet had higher perceived control (PBC) and SE (ability) towards using GFCF diet. Non-users indicated negative attitudes and outcome beliefs about using a GFCF diet, in addition to reduced perceived control and ability towards using the diet. Findings from this study are consistent with Conner et al. (2001) and Furnham and Lovett (2001). Interestingly results suggest that some parents who use GFCF diets may not be highly influenced by others’ opinions on whether they should use the diet or not, with users and non-users indicating pressure not to use GFCF diets.

Non-users of GFCF diets within this study expressed greater inhibiting factors towards using GFCF diets. Users overall perceptions about control and ability over using GFCF diets were higher and more positive compared to non-users suggesting that parents may place increased emphasis on their beliefs about possible outcomes and their own ability to inform their decisions about whether to use the diet. Wong (2009) indicated that parents chose CAM for their child with ASD as they believed it would improve the quality of their life. Furnham and Lovett (2001) similarly reported that positive outcome beliefs and attitudes distinguished users and non-users of homeopathy in relation to symptom relief. Non-users of GFCF diets may therefore be more influenced by significant others who think that they should not use the diet, believe they lack the necessary control and ability in order to implement the diets, coupled with increased negative beliefs about the possible outcome of the diet.

When examining beliefs about the causes and treatment of ASD, parents had stronger beliefs in *brain abnormalities*, *diets* and *genes and drugs*. This is partly consistent with Dardennes et al. (2011) who reported that parents believed more strongly in *brain abnormality* causes and few believed in *pregnancy and environmental* causes. In contrast to Dardennes et al. (2011), parents rated *diet* as the second highest causal factor, which was also the only significant predictor of high intentions to use GFCF diets. Increased beliefs in *diet* and *pregnancy and environmental* causal factors were also associated with the use of GFCF diets. It is widely accepted that there is a strong genetic contribution to the aetiology of ASD (Geschwind 2011) with multiple genes involved in the pathogenesis (Canitano 2013). Despite the evidence for a genetic link and there being no empirical evidence for dietary causes and treatments, parents within this study’s sample scored similarly on causal and treatment beliefs about *diet* and *genes and drugs* (along with *brain abnormalities*). From this study it is apparent that there is a requirement to provide parents of children with ASD with up-to-date information on the causes of ASD, particularly as causal beliefs of parents may influence treatment choice.

Behavioural beliefs about the use and outcome of GFCF diets was the only factor to correlate positively with their direct measure of attitude, thus positive outcome beliefs of GFCF diets are positively related to attitudes, supporting Furnham and Lovett (2001). Results are not, however, consistent with their finding of a positive relationship between normative beliefs and control beliefs with their direct measures, subjective norm and PBC. This may reflect differences in the validity of these two constructs or that the salient normative and control beliefs were not applicable towards this group of parents, as CAM use is based upon very individual cognitions. As normative beliefs and subjective norm were both independently correlated with intentions to use GFCF diets but were not related together, may suggest that the two constructs influence intentions independently. The motivation to comply component of normative beliefs (expectancy-value equation) may also supresses the subjective norm-to-normative belief-correlation (expectancy-value composite score) due to a variety of factors (Gagne and Godin 2000). One factor may be social desirability biasing, leading the individual to indicate that they are not influenced by others (Gagne and Godin 2000) (supported by the near zero score for normative beliefs). The scoring procedure for control beliefs may also impact upon its correlation with the direct measure, as a unipolar scale for both control and power items of control beliefs often yield higher correlations in addition to using only one arm of the control belief equation (control items only) (Gagne and Godin 2000). Gagne and Godin (2000) acknowledge, however, that losing one arm of the expectancy-value model results in a loss of information for differentiating between those with intention and those without. Armitage and Conner (1999a) indicated that the expectancy-value theory is weak for beliefs underlying attitude and subjective norm. They reported that the effectiveness of this equation model varied between beliefs (behavioural, normative, control) and between behaviours in question and may be useful in some contexts over others. Moreover, Armitage and Conner (1999a) provided evidence that control beliefs are only weakly associated with PBC and may be determined by other beliefs when used in a model to predict food-choice behaviour.

As predicted, an increase in positive attitudes, perceived pressure to use GFCF diets and increased feelings of anticipated regret about not using the diet was positively correlated with intentions to use GFCF diets. PBC and SE did not significantly correlate with intention, yet SE was positively correlated with PBC. This does not support a distinction between PBC and SE, and may be measuring the same construct. PBC has been argued to be over simplistic and the PBC items have weak internal reliability (Armitage and Conner 2001). Within this study, PBC’s reliability was marginally satisfactory, thus items may not have been consistently measuring PBC. These results support the utility of the TPB’s application to intentions of GFCF diet use. Interestingly, the indirect measures of attitude (behavioural beliefs) were positively correlated with intentions. Stronger positive outcome beliefs in the diet may therefore be adequate to predict intentions to use GFCF diets alone. Although intentions may not lead to actual behaviour, they are often successful in predicting behaviour (Furnham and Lovett 2001).

The results reported from the logistic regression did not however fully support the correlations. The only significant predictor of higher intentions to use GFCF diets was anticipated regret. This was independent of perceptions of control and ability over administering the diet, and perceived pressure from significant others to use GFCF diets. This does not support the hypotheses that SE, PBC, attitudes and subjective norm will emerge as predictors of intentions and is not comparable with the CAM literature, whereby intentions were predicted by positive attitudes, higher PBC and SE in the use of homeopathy (Conner and White 2009; Furnham and Lovett 2001), nor that attitude is the stronger predictor of intentions (Conner et al. 2001). When indirect measures were included in the regression the association of anticipated regret was no longer significant. Anticipated regret may be an additional predictive factor in parents’ decisions to use GFCF diets but individual beliefs about possible outcomes of the diet, the value placed on significant others beliefs about the use of GFCF diets and beliefs about inhibiting factors to use the diet may mediate this relationship. In addition, the TPB predicts that intentions and PBC can predict actual behaviour. When exploring whether GFCF diet use was predictable prospectively, the only significant predictors of users of GFCF diets were intentions and PBC. Perceptions of control may therefore be important for actual use of GFCF diets, and GFCF diets may not be under direct volitional control therefore requiring increased investment in time and resources.

Further analysis revealed the behavioural beliefs about positive outcomes of the GFCF diets of improved GI symptoms and mood were related to increased intentions to use GFCF diets. Beliefs relating to a worsening in diet variety and introducing conflict were negatively correlated with intentions, suggesting that increased negative outcome beliefs led to decreased intentions to use the diet. Parents may place emphasis on the cost-to-benefit ratio when considering GFCF diets and place emphasis on the overall perceived outcome (Furnham and Lovett 2001; Wong 2009). When the behavioural beliefs about GFCF diet outcomes were correlated with their direct measures, a similar pattern was obtained. Improved GI symptoms, mood and diet variety was associated with an increase in positive attitude. Introducing conflict and increased time necessary to implement the diet were correlated with a decrease in positive attitude. This further supports the notion that parents consider the cost-to-benefit analysis of administering a GFCF diets and particular positive behavioural beliefs about outcome may predict intentions alone when an individual feels strongly about the outcome. Previous literature has indicated that users of CAM were influenced by perceived health benefits and CAM was used for GI symptoms in children with ASD (Conner et al. 2001; Conner and White 2009; Furnham and Lovett 2001; Wong and Smith 2006). Therefore parents may also be more inclined to use GFCF diets when they believe that the diet may relieve symptoms thought to be associated with ASD.

The behavioural beliefs about the outcome that a GFCF diet worsening their child’s diet variety and introducing conflict with their child, may reduce positive attitudes about outcomes of the diet, and overall reduce intentions to use them. A belief that implementing a GFCF diet would require increased time allocation was correlated with a reduced positive attitude, but it was not correlated with a decrease in intentions, suggesting that this may have lesser influence on actual behaviour. Christon et al. (2010) note that whether parents choose to continue or discontinue with any form of CAM, is ultimately influenced by the value parents place on the treatment, which may be affected by any number of variables. Those treatments whereby parents invest more time and money may be more likely to continue (Mudford et al. 2000) and parents may feel that their child will benefit; however this, in turn, may magnify placebo effects on the efficacy of the treatment. Although the results of the correlations for behavioural beliefs are not consistent with the regression analysis whereby attitudes and behavioural beliefs were not predictors of higher intentions to using GFCF diets, dichotomising a continuous variable for logistic regression may have reduced the power of the analysis (Streiner 2002) or multicollinearity may have affected the regression. However, any correlations between variables were not unduly high.

Despite this study finding no significant correlation between the overall composite score of normative beliefs with subjective norm, when examining individual significant others, an approval to use GFCF diets from significant others (e.g. friends, family) indicated that all referents were positively related to feelings of pressure to use GFCF diets; therefore these groups of individuals may influence decisions on whether to use GFCF diets. Family (strongest correlation), friends and professionals (medical, educational) were significantly related to an increase in intentions to use the diet thus may be the most influential. Although these findings may have been due to using the belief item only of normative beliefs, previous research has highlighted the importance of friends and family in CAM (e.g. Christon et al. 2010; Furnham and Lovett 2001; Senel 2010) including GFCF diets (Cornish 2002). Conner and White (2009) also reported that those intending to use CAM were more likely to believe that their family, friends and medical/educational professionals would approve of them using CAM. Similarly to Conner and White (2009), this current study found that professionals’ approval of GFCF diet use was related to positive intentions to use the diet. When examining users and non-users, neither group expressed feelings of social pressure to use the GFCF diet, however, this may reflect a failing of transferring intentions into actual behaviour. Other factors may be important for influencing actual behaviour, such as beliefs about the value of the treatment and whether the quality of life would be improved (Christon et al. 2010; Wong 2009).

This study did not find that control beliefs (using the overall expectancy-value score) nor individual control belief items (using the control belief scoring item only), were related to intentions or PBC. The TPB states that control beliefs should not directly predict intentions but should be mediated by PBC. Users of GFCF diets were relatively neutral in their control beliefs and these factors may not have been salient for this current population of parents and did not encapsulate their beliefs. Regional variations in parent/caregiver beliefs about control factors may also yield varying findings between populations. Finally, it may also highlight a weakness in the TPB in its ability for control beliefs to reliably predict PBC (Armitage and Conner 1999b) particularly as PBC was not a significant predictor of intentions.

The results of this study provide some indication of possible predictors of intentions to use GFCF diets and actual use, namely, anticipated regret. There are several possible explanations as to why the regression analysis did not find any other TPB variable to predict intensions. Documented weaknesses within the TPB variables and their ability to predict intentions to use GFCF diets may offer some explanation. A skewed sample of parents, who did not intend to use GFCF diets and had never used them, may have been recruited. Parents overall had weak intentions and intention scores did not significantly differ between users and non-users. The direction of scores were, however, as predicted, with users indicating a trend towards higher intentions. Furthermore, as users consisted of those who had used GFCF diets in the past and current users, parents who had previously used GFCF diets may not intend to use them again for various reasons, including negative outcome beliefs and pressure from salient others. This in turn may have weakened the overall intention score for users. Although this study used the current scoring recommendations, there is no consensus on the most appropriate scoring procedure for the expectancy-value model, which may explain the non-significant findings. Other factors may also be important such as the beliefs used in this study may not apply to this group of parents and may not be stable over time (Ajzen 2002b) or between individuals and contexts.

The correlations on individual behavioural beliefs about outcome highlighted that an improvement in GI symptoms and mood may be important factors for parents with children who experience these symptoms. Worsening diet variety and introducing conflict with their child may be inhibiting factors for non-users and those with lower intentions to use GFCF diets. Approval from friends, family and professionals may influence decisions on whether to use the diet or not. Professionals may also have an impact upon treatment choice and approval. Further study should gather information which facilitates parent’s need for professional approval.

The results of this present study offer a unique glimpse into the factors relating to parents’ intentions and use of GFCF diets. Despite the lack of empirical support for GFCF diets, parents may need further information regarding this type of CAM. Targeting particular parent support groups may provide an intervention focus, particularly as parents regard the approval of friends and family. Conventional medical providers should also provide information on the current recommendations for therapies and interventions in addition to informing parents about those therapies and treatments that are not scientifically supported. Parents, who hold strong beliefs about the causes of autism in relation to *diet* and *brain abnormalities,* may hold unrealistic outcome beliefs about the GFCF diet. Parents suggested that a reduced diet variety and conflict with their child were factors which led to decreased intentions and these factors may outweigh perceived benefits of the GFCF diet. Further investigation of the cost-to-benefit analysis rated by parents should be examined to ensure that interventions target salient beliefs which outweigh the costs of the diet.

Parents within this study did not detail other CAM their child may use. Senel (2010) reported that parents often try multiple CAM treatments simultaneously. Therefore parents’ beliefs may have been unintentionally influenced by the other forms of CAM used. Christon et al. (2010) also note that when making medical decisions, people may be susceptible to cognitive biasing by aligning the probabilities of outcomes and altering them to fit their own perceptions or desire, which consequently bias judgments on efficacy. This may distort parents’ perceptions about treatment to avoid cognitive dissonance. Conner and Sparks (2005) also note that the TPB does not account for other non-cognitive or irrational determinants of human behaviour such as emotion. The topic of ASD, interventions and causal beliefs is a highly emotive subject and it is unclear whether emotional factors interplay with cognitions. Further research should examine how emotionality may be controlled for.

## Conclusion

This is the first study that addresses cognitions of parents about GFCF diets for children with ASD using a theoretical framework and good reliability in the measure used. Parents were guided by positive outcome beliefs and assess the cost-to-benefit analysis of GFCF diets when considering use. Parents may be guided by feelings of regret for not using the diet, with the fear that they had not tried all available interventions. Perceptions of control may be important for predicting actual use. These findings should assist with the development of interventions to bring parents’ expectations and outcome beliefs about GFCF diets in-line with current evidence and guidelines. Parents should be informed about the current information on causal attributes and therapy options for ASD.
